# Type I-E* CRISPR-Cas of *Klebsiella pneumoniae* upregulates bacterial virulence by targeting endogenous histidine utilization system

**DOI:** 10.1128/msphere.00215-25

**Published:** 2025-05-19

**Authors:** Jieying Li, Yuxiao Liu, Jingsi Jiang, Fang Chen, Nan Zhang, Xun Kang, Lin Liu, Yingjuan Wang, Qianfeng Xia, Chuanlong Zhu, Dai Kuang

**Affiliations:** 1 NHC Key Laboratory of Tropical Disease Control, School of Tropical Medicine, Hainan Medical University 12455, Haikou, Hainan, China; 2 Department of Infectious and Tropical Diseases, The Second Affiliated Hospital of Hainan Medical University 477165, Haikou, Hainan, China; 3 School of Hainan Provincial Drug Safety Evaluation Research Center, Hainan Medical University 12455, Haikou, Hainan, China; 4 Laboratory of Infectious Disease, The First Affiliated Hospital of Nanjing Medical University 74734, Nanjing, Jiangsu, China; University of Kentucky College of Medicine, Lexington, Kentucky, USA

**Keywords:** CRISPR-Cas system, *Klebsiella pneumoniae*, virulence, self-targeting regulation, histidine utilization

## Abstract

*Klebsiella pneumoniae* is a globally recognized microbial pathogen with significant clinical impact. The bacterium harbors the clustered regularly interspaced short palindromic repeats (CRISPR)-Cas systems, which provide adaptive immunity against invading foreign nucleic acids. Recent studies suggest that certain CRISPR-Cas systems can regulate endogenous genes, influencing bacterial virulence. However, their role in regulating pathogenicity in *K. pneumoniae* remains poorly understood. This study investigates the regulatory role of the type I-E* CRISPR-Cas system in a hypervirulent *K. pneumoniae* strain, focusing on its impact on histidine metabolism and pathogenicity. Transcriptome analyses identified differentially expressed genes (DEGs) between the *casABECD*-deletion and wild-type strains, including significant upregulation of the histidine utilization (Hut) operon and downregulation of biofilm-related genes. These molecular changes resulted in enhanced histidine metabolic activity, reduced biofilm formation, attenuated virulence in A549 lung epithelial cells, and improved survival of *Galleria mellonella*, as validated through phenotypic and virulence assays. Our bioinformatic analysis indicated that the CRISPR-Cas system in *K. pneumoniae* targets the *hutT* sequence, which is part of the Hut operon. Furthermore, the overexpression of *hutT* mitigated CRISPR-Cas-mediated repression of the Hut operon, as observed in virulence assays, while simultaneous deletion of *hutH* and *casABECD* restored the reduced virulence in the Δ*casABECD* strain. Additionally, deletion of *casABECD* significantly enhances the growth of the strain in medium with histidine as the sole carbon source, highlighting the intricate regulatory role of the CRISPR-Cas system in metabolic adaptation. Collectively, these findings uncover a novel role for the CRISPR-Cas system in regulating metabolic pathways and virulence in hypervirulent *K. pneumoniae*.

**IMPORTANCE:**

Clustered regularly interspaced short palindromic repeats (CRISPR)-Cas systems are primarily recognized for their roles in adaptive immunity against foreign genetic elements in bacteria. However, emerging evidence indicates that these systems can also regulate endogenous genes, thereby influencing bacterial physiology and virulence. In this study, we demonstrate that the type I-E* CRISPR-Cas system in *Klebsiella pneumoniae* targets the *hutT* gene, a critical component of the histidine utilization (Hut) pathway. This targeting potentially impacts *hutT* transcription and alters the expression of other *hut* genes, ultimately enhancing bacterial virulence. Our findings reveal a previously unrecognized regulatory mechanism through which CRISPR-Cas systems facilitate metabolic adaptation and pathogenicity in *K. pneumoniae*. This study broadens our understanding of the multifaceted roles of CRISPR-Cas systems in bacterial physiology and pathobiology, with implications for clinically relevant pathogens.

## INTRODUCTION

*Klebsiella pneumoniae* (*K. pneumoniae*, Kp) is a globally recognized microbial pathogen, ranking as one of the most prevalent causes of clinical infections, second only to *Escherichia coli* in China (1). Traditionally, *K. pneumoniae* has been classified into two distinct pathotypes (2, 3): hypervirulent Kp (hvKp), which is associated with severe invasive infections in otherwise healthy individuals, and classical Kp (cKp, non-hvKp), which predominantly causes hospital-acquired infections and is often multidrug resistant (MDR), leaving limited therapeutic options (4). In Asia, the most prevalent MDR-Kp variant is multilocus sequence type (ST) 11, which belongs to clonal group (CG) 258, whereas ST23 is the predominant clone of hvKp (5, 6). However, the boundaries between hvKp and cKp are becoming increasingly ambiguous (7), raising concerns about the potential emergence of *K. pneumoniae* as a “superbug” due to the convergence of virulence and resistance traits (8).

Clustered regularly interspaced short palindromic repeats (CRISPR) and their associated proteins (CRISPR-associated, Cas) provide prokaryotes with adaptive immunity by recognizing and eliminating foreign nucleic acids, including those from phages, plasmids, and transposons (9). The canonical role of CRISPR-Cas systems in protecting against mobile genetic elements (MGEs) has been extensively studied and has led to the development of CRISPR technology, a revolutionary molecular tool (10). Beyond this established function, growing evidence suggests that CRISPR-Cas systems also participate in processes such as gene regulation, bacterial pathophysiology, virulence, and evolution (11–14). The CRISPR array incorporates foreign DNA fragments as spacers for future recognition and elimination. These spacers are transcribed to produce CRISPR RNA (crRNA), which guides the system (15). Interestingly, some spacers originate from the host’s genomic DNA and may be involved in regulatory processes (16). For example, in *Francisella novicida*, the type II CRISPR-Cas system regulates the expression of a virulence-associated protein-coding gene (12, 17). Similarly, in *Pelobacter carbinolicus*, a self-targeting spacer was found to downregulate histidyl-tRNA synthetase (*hisS*) expression (18).

Comparative genomic studies have categorized the CRISPR-Cas systems in *K. pneumoniae* into two types: subtype I-E and subtype I-E*, based on Cas1 and Cas3 protein and chromosomal localization (19). These systems are more prevalent in hvKp isolates and less common in MDR-Kp isolates (19–21), suggesting divergent evolutionary paths: hvKp strains have retained CRISPR-Cas systems for functions yet to be fully elucidated, while MDR-Kp strains may have lost them to facilitate the acquisition of exogenous antibiotic resistance genes. Notably, plenty of hvKp strains, including the infamous NTUH-K2044 and ATCC 43816, harbor the I-E* subtype CRISPR-Cas system. Furthermore, a high proportion of self-targeting spacers has been identified in CRISPR-bearing *K. pneumoniae* isolates (19), suggesting a potential role in regulating endogenous genes that could influence virulence.

In this study, we investigated the role of the type I-E* CRISPR-Cas system in hvKp by analyzing a wild-type (WT) hvKp strain, Kp674, isolated from a hospital patient. Kp674 belongs to the ST23 subtype, exhibits the K1 capsular serotype, and harbors the I-E* CRISPR-Cas system. To explore the functional role of this system in virulence regulation, we constructed a CRISPR-associated complex for antiviral defense (Cascade) subunit region deletion mutant (Δ*casABECD*) of Kp674. We then compared the WT and Δ*casABECD* strains by assessing key virulence phenotypes, such as biofilm formation, cytotoxicity, and *Galleria mellonella* infection assays. Furthermore, transcriptome analysis, CRISPR-Cas system target identification, and related corroborative experiments were performed to identify genes regulated by the CRISPR-Cas system. Our findings demonstrate that the type I-E* CRISPR-Cas system upregulates bacterial virulence primarily through targeting histidine utilization (Hut) systems.

## RESULTS

### Deletion of *casABECD* does not affect bacterial growth

To investigate the influence of the CRISPR-Cas system on endogenous gene regulation and virulence in hvKp, we generated the *casABECD* gene deletion strain Δ*casABECD* and the complemented strain C-*casABECD*, starting from the WT ST23 hvKp strain Kp674.

**Fig 1:**
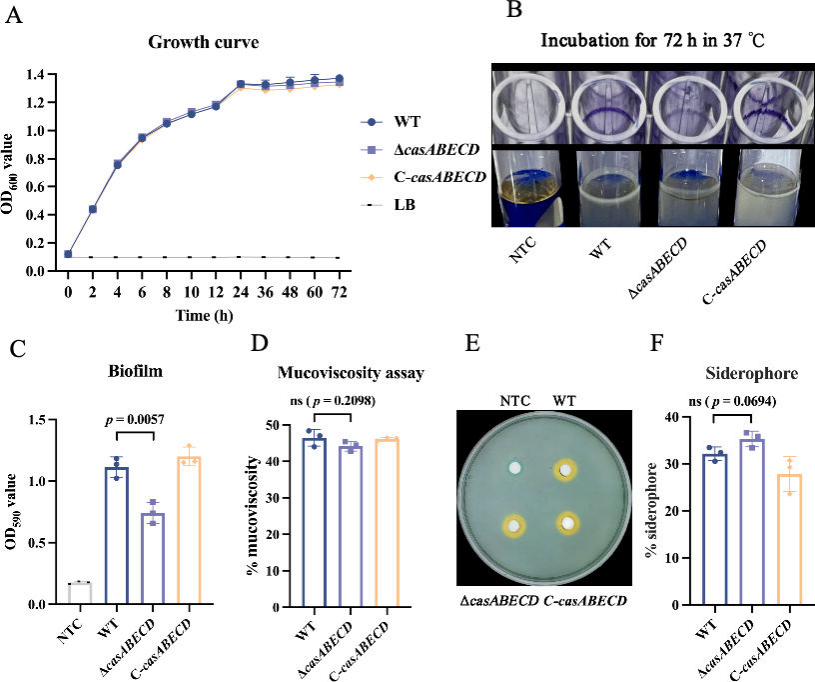
Impact of *casABECD* on bacterial growth and virulence-related traits. (**A**) Deletion of *casABECD* has no effect on bacterial growth in LB broth. (**B**) Visualization of biofilm formation in the WT, Δc*asABECD*, and C-*casABECD* strains after 72 h of incubation at 37℃ under static conditions using the crystal violet and test tube methods. (**C**) Quantification of biofilm formation in LB broth using the crystal violet staining method. (**D**) Measurement of bacterial mucoviscosity by low-speed centrifugation in LB broth. (**E**) Siderophore production visualized on CAS agar plates, with an orange secretion ring around the colony, indicating siderophore generation. (**F**) Deletion of *casABECD* does not affect bacterial siderophore generation (*P* > 0.05). NTC, non-treated control; bars, mean ± SD, *n* = 3.

We then assessed the effect of the *casABECD* gene deletion on bacterial growth in Luria-Bertani (LB) broth at 37°C by determining standard growth curves (Fig. 1A). The results indicated that there were no statistically significant differences in the growth of the WT, Δ*casABECD*, and C-*casABECD* strains, suggesting that the deletion of the *casABECD* gene had no impact on bacterial viability.

### Deletion of *casABECD* reduces bacterial biofilm formation

Given the relevance of biofilm formation, capsule production, and siderophore production to hvKp virulence (22), we evaluated the role of *casABECD* in these traits. The WT, Δ*casABECD*, and C-*casABECD* of Kp674 strains all exhibited detectable biofilm formation on the plastic surface, visualizing as a floating pellicle and a ring of bacteria adhered to the tube wall at the air–liquid interface (Fig. 1B) (23). However, the Δ*casABECD* strain showed significantly reduced biofilm formation compared to the WT strain, a phenotype that was reversed by in *trans* complementation (Fig. 1C).

K1 capsular serotype *Klebsiella pneumoniae* is typically characterized by hypermucoviscosity (HMV), which represents a critical phenotype of hypervirulence (24). Thus, mucoviscosity was quantified by low-speed centrifugation, where HMV strains remain buoyant, leading to a turbid supernatant. The results showed no statistically significant differences in mucoviscosity between the WT, Δ*casABECD*, and C-*casABECD* strains (Fig. 1D). Furthermore, siderophore production was qualitatively assessed using the Chromazurol S (CAS) assay, which showed that all strains produced orange halos on CAS plates (Fig. 1E). Quantitative analysis of siderophore production revealed no significant differences between the WT, Δ*casABECD*, and C-*casABECD* strains (Fig. 1F).

### Deletion of *casABECD* reduces bacterial virulence in A549 cells and *Galleria mellonella* larvae

Our results indicate that the *casABECD* gene is involved in the regulation of biofilm formation, which is considered a relevant virulence factor for hvKp (25). Thus, to investigate the effect of *casABECD* deficiency on virulence, we employed non-small lung carcinoma A549 cells and *Galleria mellonella* larvae as model hosts, which have been widely used and validated for assessing the virulence of *K. pneumoniae* (26, 27).

**Fig 2:**
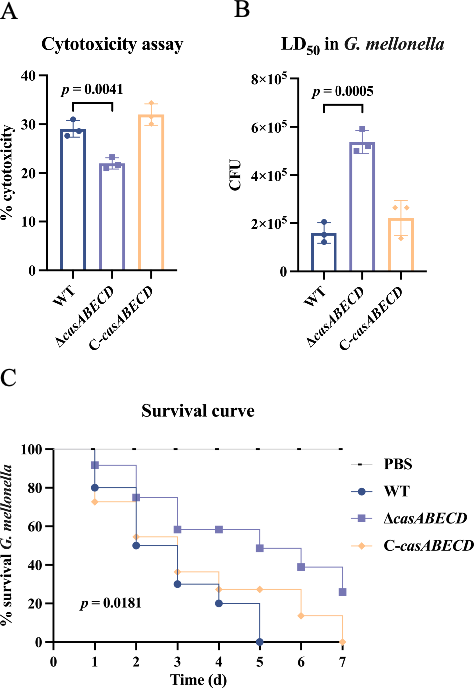
Impact of *casABECD* on the bacterial virulence on A549 cells and *Galleria mellonella* larvae. (**A**) LDH release from A549 cells after 4 h of infection with the WT, Δ*casABECD*, and C-*casABECD* strains at a 100:1 MOI. (**B**) LD_50_ of *K. pneumoniae* strains in *G. mellonella* larvae at 72 h postinfection. (**C**) Survival of *G. mellonella* larvae injected with PBS or 5 × 10^5^ CFU of *K. pneumoniae* monitored for 7 d postinfection. Bars, mean ± SD, *n* = 3.

Bacteria were added at a multiplicity of infection (MOI) of 100:1 per cell and were incubated for 4 h. In the lactate dehydrogenase (LDH) release assays, A549 cells infected with the Δ*casABECD* strain exhibited reduced LDH release and a lower number of dead cells compared to those infected with the WT strain; this phenotype was restored by in *trans* complementation (Fig. 2A). Furthermore, the *G. mellonella* larvae were injected with various dilutions of different strains, ranging from 10^4^ to 10^6^ CFU, and mortality was monitored over 72 h. The 50% lethal dose (LD_50_) for the Δ*casABECD* strain was significantly higher than for the WT strain, with the phenotype reversed by in *trans* complementation (Fig. 2B). Larvae were injected with phosphate-buffered saline (PBS) or 5 × 10^5^ CFU of bacteria, and survival was monitored over a 7 d period to generate survival curves. In the *G. mellonella* infection model, the WT strain killed approximately 50% of the larvae within 48 h, compared to 30% mortality observed in larvae injected with Δ*casABECD* (Fig. 2C). This difference persisted throughout the 7 d observation period. As a control, all larvae in the PBS group survived. These results suggest that the deficiency of *casABECD* in *K. pneumoniae* diminishes its virulence toward *G. mellonella*.

### Transcriptomic analysis reveals differential gene expression between wild-type and Δ*casABECD* strains

**Fig 3:**
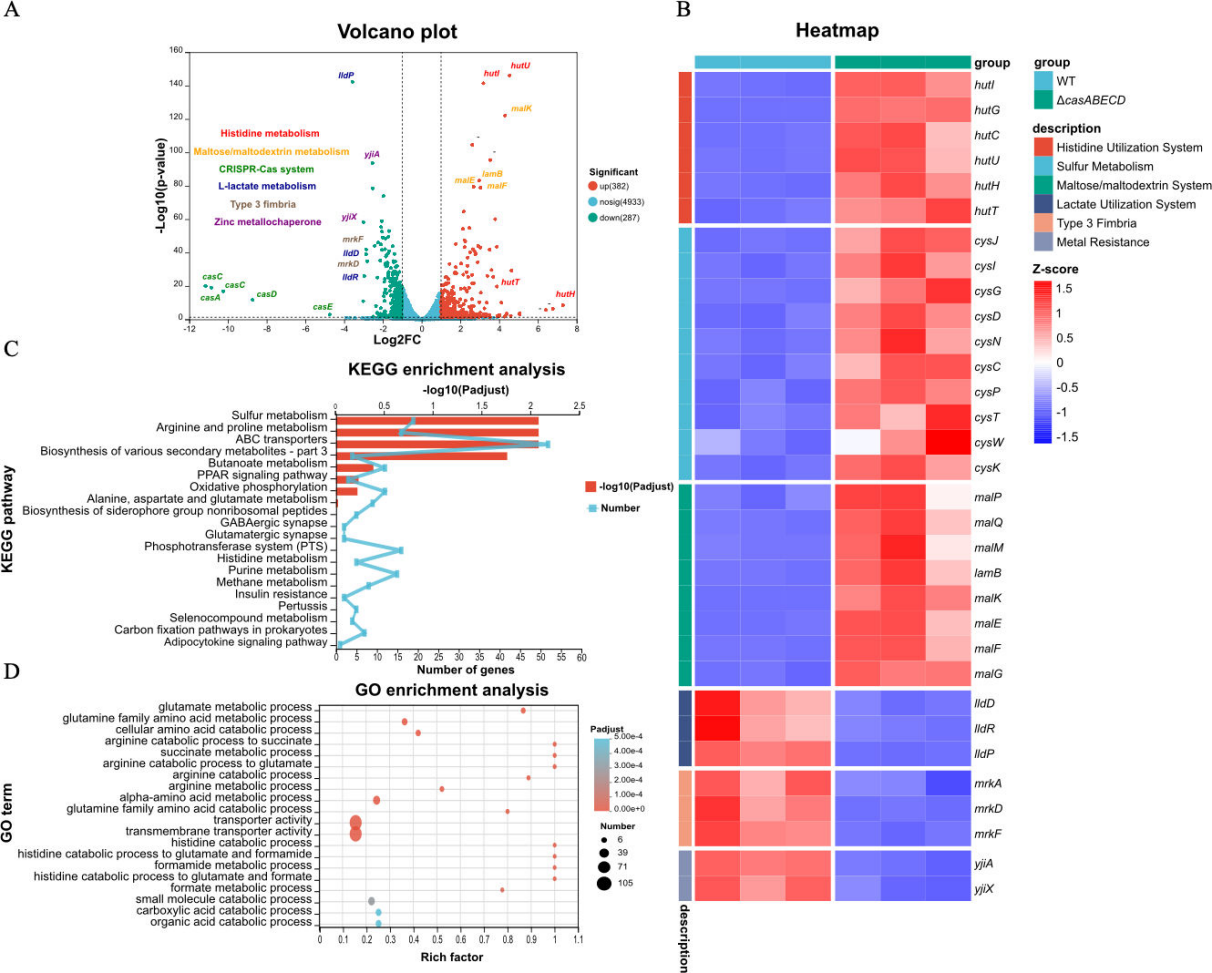
Transcriptomics profiling of *casABECD* mutants in *K. pneumoniae*. (**A**) Volcano plot of DEGs in the *casABECD* mutants. These colored points represent different genes with large fold changes (log_2_ of fold changes, *x*-axis) and high statistical significance (–log_10_ of *P* values, *y*-axis). Functional groups are color coded. (**B**) The heatmap shows selected important DEGs in *casABECD* mutants. Gene expression levels are displayed in transcripts per million (TPM), normalized by row-wise Z-score transformation to highlight changes across samples. Z-scores are mapped to a color scale, with red indicating upregulation and blue indicating downregulation. (**C**) KEGG enrichment analysis. The top axis shows –log_10_ (”Padjust”). The bottom axis displays the number of enriched genes. (**D**) GO enrichment analysis. The *x*-axis represents the rich factor (ratio of DEGs annotated to a GO term vs total genes annotated to that term). The number indicates enriched gene counts per term. Padjust indicates the adjusted *P* value.

To explore the underlying molecular mechanisms responsible for the observed reduction in biofilm formation and virulence, we performed RNA seq and transcriptomic analysis by comparing the Δ*casABECD* to the wild-type strain. A total of 669 genes were differentially expressed, including 382 upregulated and 287 downregulated genes in the Δ*casABECD* strain. The differential expression levels of these genes are presented in a volcano plot (Fig. 3A).

To further analyze the differentially expressed genes (DEGs), we selected those with the most significant *P* values and fold changes, examined the gene clusters to which they belong, and visualized the important DEG groups together in a heatmap (Fig. 3B). Among the most significant DEGs, several genes in the Hut operon (*hutI*, *hutG*, *hutC*, *hutU*, *hutH*, and *hutT*), which are involved in the histidine utilization system (28), were markedly upregulated. Additionally, the expression of several biofilm-associated genes (*mrkA*, *mrkD*, and *mrkF*), which are linked to type III fimbriae, was significantly decreased (29). In addition, other important DEG groups include sulfate metabolism-related genes, metal resistance, the periplasmic maltose-binding protein of ABC transporters (30), and *L*-lactate transport genes related to colonization (31).

Then, DEGs were mapped to the Kyoto Encyclopedia of Genes and Genomes (KEGG) and Gene Ontology (GO) databases, and linked to important pathways and functions based on the entire transcriptome background. The KEGG enrichment analysis showed the four pathways that were significantly enriched, shown in Figure 3C, namely, “sulfur metabolism,” “arginine and proline metabolism,” “ABC transporters,” and “biosynthesis of various secondary metabolites—part 3.” The GO enrichment analysis revealed the top 20 significantly enriched GO terms. Terms with the highest enrichment factors were primarily associated with arginine and histidine catabolism. Terms with the highest number of enriched DEGs were linked to transporter and transmembrane transporter activities, aligning with the ABC transporter pathway from KEGG, further supporting the role of DEGs in substance transport (Fig. 3D).

### Specific *hutT* sequences are targeted by type I CRISPR-Cas systems

**Fig 4:**
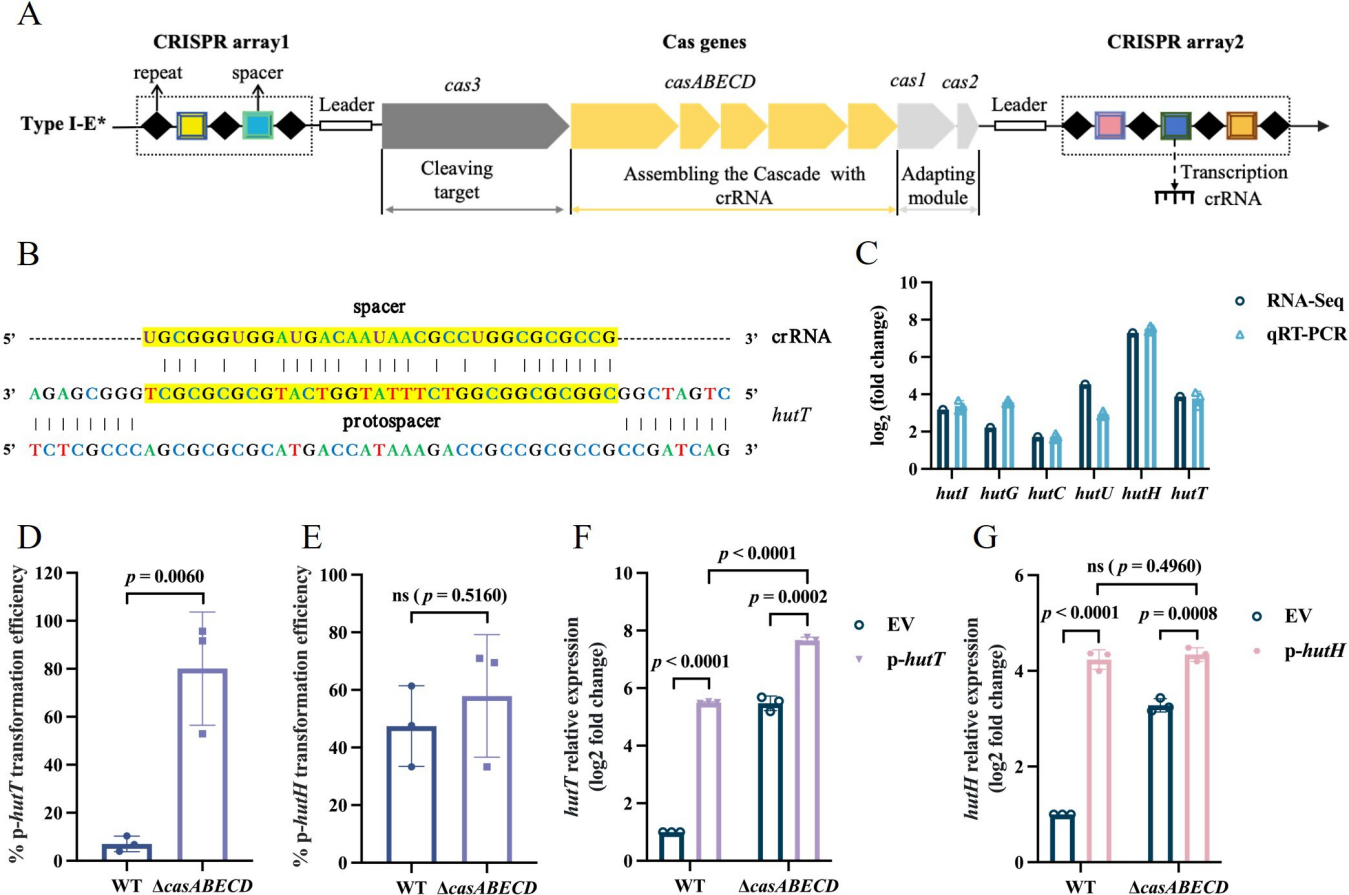
Type I-E* CRISPR-Cas of *K. pneumoniae* targets the histidine transporter *hutT*. (**A**) Schematic of Kp674 type I-E* CRISPR-Cas locus, comprising two CRISPR arrays and eight *cas* genes that are arranged as *cas3-casA-casB-casE-casC-casD-cas1-cas2*. (**B**) Homology comparison between *hutT* DNA and spacer. The protospacer is the DNA target complementary to the crRNA spacer. The crRNA is displayed as RNA 5’ to 3’, and the base-paired protospacer is 3’ to 5’. Yellow sequences include spacer and protospacer. (**C**) Comparison of the expression level of *hut* operon genes acquired from RNA seq and qRT-PCR. (**D**) Transformation efficiency was determined by colony counts in introducing the p-*hutT* or the EV into the WT and Δ*casABECD* strains. (**E**) Transformation efficiency was determined by colony counts after introducing the p-*hutH* or the EV into the WT and Δ*casABECD* strains. (**F**) *hutT* transcript levels in the plasmid-transformed WT and Δ*casABECD* strains were quantified by qRT-PCR. (**G**) *hutH* transcript levels in the plasmid-transformed WT and Δ*casABECD* strains were quantified by qRT-PCR. Data are representative of three independent experiments and expressed as means ± standard error of the mean (SEM) (*n* = 3).

CRISPRCasfinder identified that Kp674 carries an I-E* type CRISPR-Cas system with two CRISPR arrays, including a total of 50 spacers (Fig. 4A) (Table S1). CRISPR-Cas systems have been shown to target endogenous bacterial genes, thereby modulating virulence and potentially influencing host–pathogen interactions; however, such roles have not been documented in *K. pneumoniae*. We used CRISPRTarget to detect the targets of crRNAs in Kp674’s own sequences. Sequence alignment revealed substantial homology between the *hutT* gene and CRISPR crRNA spacers, although no protospacer adjacent motif (PAM) sequence was detected (Fig. 4B). Based on bioinformatics analysis of CRISPR-Cas systems in 1,194 fully sequenced *Klebsiella pneumoniae* from a previous study (32), we further examined spacers targeting the *hutT* gene (Table S2). Notably, the results showed that 21 strains harbored spacers matching the *hutT* DNA, including 18 ST23 strains (42.9% of all ST23 strains) and three non-ST23 strains (two ST1941 and one ST1660) (Table S2), suggesting the importance of genetic background in spacer distribution. To validate the RNA-seq findings, we performed qRT-PCR analysis normalized using 16S rRNA as the internal reference gene and confirmed that the expression of *hut* genes in the Δ*casABECD* strain was significantly upregulated. These results were consistent with the RNA-seq data (Fig. 4C).

To determine whether the Kp674 CRISPR-Cas system targets *hutT*, artificially simulated exogenous *hutT*-harboring plasmid invasion assays were performed in the WT and Δ*casABECD* strains. The results showed that the Δ*casABECD* strain displayed higher colony-forming efficiency upon plasmid p-*hutT* transformation (Fig. 4D), whereas no significant difference was observed with the CRISPR non-targeting control plasmid (p-*hutH*) transformation (Fig. 4E), indicating that the Kp674 CRISPR-Cas system does target *hutT* DNA. Specifically, the CRISPR system uses spacer sequences to target plasmids containing the *hutT* gene, thereby affecting plasmid transformation efficiency. Additionally, exogenous plasmid p-*hutT* significantly induced *hutT* expression in both the WT and Δ*casABECD* mutant strains, but *hutT* expression level in the WT was much lower than that in the Δ*casABECD* strain (Fig. 4F), suggesting that the Kp674 CRISPR-Cas can also repress exogenous *hutT* expression. This repression was spacer targeting dependent, as no significant difference was observed with the non-targeting p-*hutH* plasmid control (Fig. 4G).

Combining RNA-seq analysis and CRISPRTarget analysis, we found that the *hut* gene cluster is upregulated together after *casABECD* gene deletions. Among 384 upregulated genes, the *hutH* gene showed the largest fold change, while the *hutU* gene showed the smallest *P* value. Notably, crRNA targets the *hutT* DNA sequence, which is adjacent to the *hutU* and *hutH* genes on the chromosome, with the *hutUH* genes forming an operon in *K. pneumoniae* (33). The position of *hutT* relative to *hutUH* suggests the potential existence of a *hutUHT* operon, and the activity of HutT is typically co-regulated with HutH (34). Therefore, we hypothesized that the CRISPR-Cas system modulates virulence by primarily targeting the *hut* gene cluster in Kp674.

### CRISPR-Cas system modulates virulence in *K. pneumoniae* via regulation of the *hut* gene cluster

**Fig 5:**
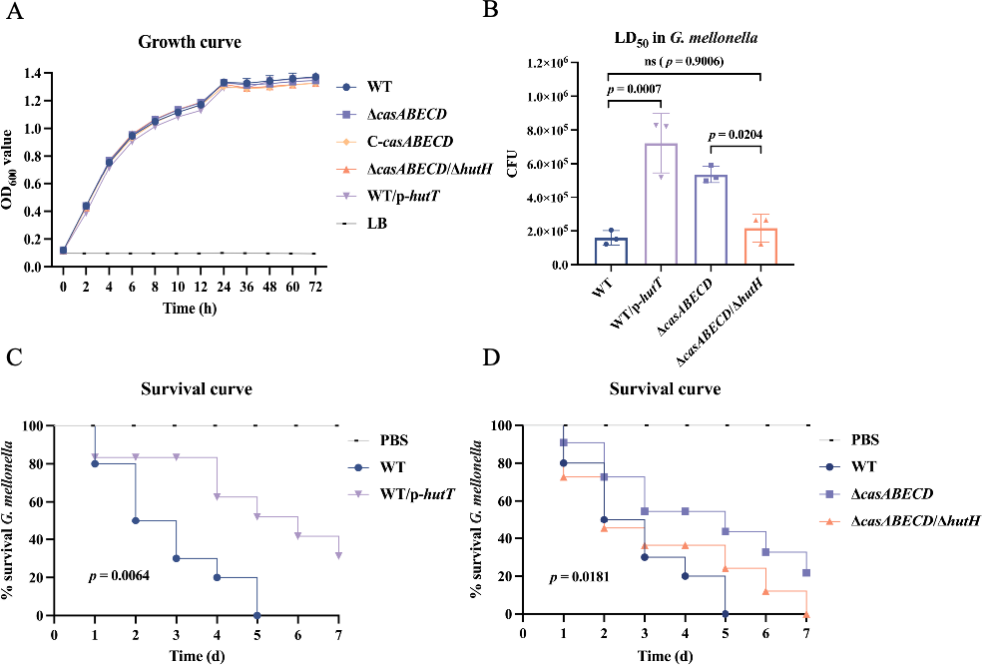
HutH and HutT contribute to *K. pneumoniae* pathogenesis. (**A**) Deletion of *casABECD* and *hutH*, or complementation with plasmids, does not affect bacterial growth in LB broth under standard conditions (*P* > 0.05). (**B**) LD_50_ of *K. pneumoniae* strains in *G. mellonella* larvae at 72 h postinfection. (**C**) Survival of *G. mellonella* larvae injected with PBS or 5 × 10^5^ CFU of the WT and WT/p-*hutT* strains, monitored for 7 d postinfection. (**D**) Survival of *G. mellonella* larvae injected with PBS or 5 × 10^5^ CFU of the WT, Δ*casABECD*, and Δ*casABECD*/Δ*hutH* monitored for 7 d postinfection. Data are representative of three experiments expressed as means ± SEM (*n* = 3, one-way ANOVA with Tukey’s post hoc); Kaplan-Meier survival curves were compared using a log-rank test.

To investigate whether the deletion of *casABECD* leads to the overexpression of endogenous histidine utilization system genes and thereby impacts bacterial virulence, we constructed a double-deletion strain lacking both *casABECD* and *hutH* (Δ*casABECD/*Δ*hutH*). Also, we generated an overexpression strain for *hutT* (WT/p-*hutT*) based on the wild-type strain to determine whether excessive *hutT* expression could counteract the endogenous targeting and repression exerted by the CRISPR-Cas system. Growth curve analyses in LB medium revealed no significant differences among the strains (Fig. 5A).

The LD_50_ of the Δ*casABECD*/Δ*hutH* strain was significantly lower than that of the Δ*casABECD* strain, whereas the overexpression strain WT/p-*hutT* exhibited a significantly higher LD_50_ compared to the wild-type strain (Fig. 5B). Then, *Galleria mellonella* larvae were injected with PBS or 5 × 10^5^ CFU of bacteria, and survival was monitored over 7 d postinfection to generate survival curves. Infection with the WT strain resulted in approximately 100% mortality within 5 d, whereas larvae infected with the WT/p*-hutT* strain exhibited significantly improved survival (Fig. 5C). Compared to the WT strain, infection with the Δ*casABECD* strain improved survival, a phenotype that was reversed by the Δ*casABECD*/Δ*hutH* double-deletion strain (Fig. 5D). These results suggest that the suppression of the *hut* gene cluster in the wild-type strain can be alleviated by overexpressing *hutT*, potentially because excessive *hutT* expression attracts the “targeting focus” of the Cascade complex encoded by *casABECD*.

### Deletion of *casABECD* enhances histidine utilization

The Hut pathway is essentially a histidine catabolic pathway that allows bacteria to use histidine as a source of carbon/nitrogen (28). To determine whether the deletion of the *casABECD* genes affects the expression of the *hut* gene cluster and thereby regulates histidine metabolism, we performed histidine utilization assays. In these assays, bacterial cultures were diluted 1:100 in M9 minimal medium containing a twofold dilution series of *L*-histidine (0–256 mM) as the sole carbon source. Growth was monitored over time to evaluate the impact of *casABECD* deletion on histidine utilization efficiency.

**Fig 6:**
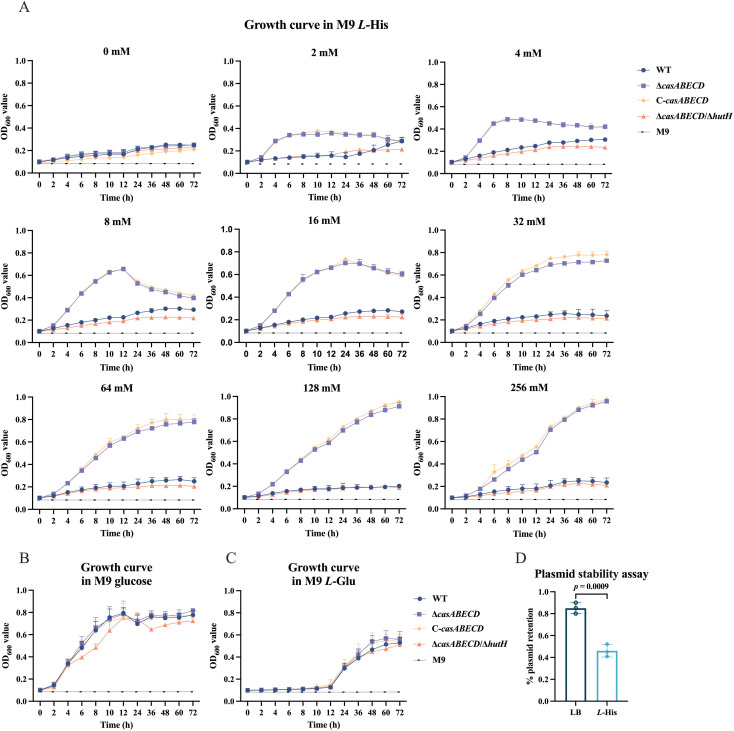
Deficiency of *casABECD* in *K. pneumoniae* enhances the bacterial ability to metabolize histidine. (**A**) Growth of the WT and mutant strains in M9 medium with varying *L*-histidine (*L*-His) concentrations. (**B**) Growth of the WT and mutant strains in M9 medium with 0.4% glucose. (**C**) Growth of the WT and mutant strains in M9 medium with 10 mM *L*-glutamate (*L*-Glu). (**D**) Plasmid retention in the C-*casABECD* strain in M9 medium with *L*-His compared to LB medium. Data are representative of three experiments expressed as means ± SEM (*n* = 3).

The results demonstrated that the growth of all tested strains was significantly restricted in minimal M9 medium without any additional carbon source (Fig. 6A). However, upon the addition of a low concentration of 2 mM *L*-histidine (*L*-His), the Δ*casABECD* strains partially resumed growth, whereas the double-deletion Δ*casABECD*/Δ*hutH* strain failed to recover growth. This trend was observed across all tested *L*-histidine concentrations (Fig. 6A), indicating that *casABECD* deletion enhances *hut* gene expression and improves histidine metabolism. In control experiments with non-histidine carbon sources, all strains showed no significant growth differences in M9 medium with either 0.4% glucose (Fig. 6B) or 10 mM *L*-glutamate (*L*-Glu) as the sole carbon source (Fig. 6C). Notably, the C-*casABECD* failed to restore the growth-restricted phenotype as expected when histidine was the sole carbon source, showing a growth capacity similar to the Δ*casABECD* strain. This observation might be attributed to the loss of the complementation plasmid under conditions where histidine is the sole carbon source, as confirmed by plasmid stability assays (Fig. 6D).

## DISCUSSION

Beyond their canonical role in adaptive immunity, CRISPR-Cas systems are increasingly recognized for their non-canonical or alternative functions in gene regulation, bacterial pathophysiology, virulence, and evolution. This study demonstrates that the type I-E* CRISPR-Cas system of *Klebsiella pneumoniae* influences virulence by targeting and repressing the histidine utilization system. These findings highlight the capacity of CRISPR-Cas systems to regulate bacterial functions by potentially self-targeting endogenous genes in *K. pneumoniae*.

The effects of CRISPR-Cas systems on virulence vary across bacterial strains. For example, Sampson et al. found that the CRISPR-Cas9 system in *Francisella novicida* targets bacterial lipoprotein mRNA, altering membrane permeability, promoting antibiotic resistance, and enhancing pathogenicity by preventing host immune recognition (12, 17). In *Salmonella enterica*, the deletion of *cas3* has been shown to reduce biofilm formation and virulence toward host cells. Transcriptome analysis revealed that *cas3* deletion affects genes related to quorum sensing, the type III secretion system (T3SS), *Salmonella* pathogenicity island-1 (SPI-1), and genes associated with flagellum formation (35). Additionally, the reduced virulence of the type I CRISPR-Cas system of *Pseudomonas aeruginosa* PA14 can be attributed to the transcriptional regulation of the quorum-sensing regulator *lasR*, which in turn inhibits recognition by the host TLR4, thereby achieving immune evasion (13). Similarly, deletion of *cas3* in *Porphyromonas gingivalis* increases virulence in THP-1 cells in the *Galleria mellonella* killing assay (36). These findings collectively suggest that CRISPR-Cas systems regulate bacterial resistance and virulence by acting on diverse target sites, paving the way for further exploration of their biological roles.

Despite these insights, the precise mechanisms through which CRISPR-Cas systems influence virulence in *K. pneumoniae* remain poorly understood. Previous studies have suggested that the absence of CRISPR-Cas systems in ST11 and ST258 *K. pneumoniae* may have facilitated the development of antibiotic resistance, as these systems inhibit the horizontal transfer of resistance plasmids (21, 32). In contrast, high-virulence strains, such as the ST23 type of *K. pneumoniae* responsible for large-scale infections, often possess the CRISPR-Cas system. For instance, Liao et al. found that isolates having the subtype I-E* CRISPR-Cas system tended to have more virulence genes and a shorter average survival time of infected *G. mellonella* larvae, compared to CRISPR-negative isolates and type I-E CRISPR-Cas isolate (37). Furthermore, Shen et al. reported a high prevalence of self-targeting spacers in CRISPR-bearing *K. pneumoniae* (19). These findings suggest a potential link between the CRISPR-Cas system and the virulence phenotype of *K. pneumoniae*, indicating that it may regulate virulence through self-targeting endogenous genes. Consistent with this hypothesis, our study demonstrated that the CRISPR-Cas system enhances the virulence of *K. pneumoniae* by comparing the cytotoxicity and *Galleria mellonella* survival rates between the ∆*casABECD* mutant and the wild-type strain.

Histidine is one of the most energy-intensive amino acids to synthesize and plays a pivotal role in bacterial metabolism and virulence (38). It is derived from 5′-phosphoribosyl-1-pyrophosphate (PRPP) and serves as a precursor for key pathways, such as purine biosynthesis (39). Any abnormal degradation or accumulation of histidine can potentially affect cellular metabolic functions and protein synthesis efficiency (38). Excess histidine is degraded through the bacterial histidine utilization system into ammonia, glutamate, and one-carbon compounds (formate or formamide) (28). The *hut* cluster in *K. pneumoniae* consists of six genes: four that encode enzymes (*hutI*, *hutG*, *hutU*, and *hutH*); *hutC*, which encodes a transcriptional regulator belonging to the GntR family; and *hutT*, which encodes the urocanate transporter (28). Through transcriptome analysis and further confirmed by qRT-PCR, we found that the expression of the *hut* gene cluster was significantly higher in the ∆*casABECD* mutant compared to the wild-type strain. Our further analysis using CRISPRTarget indicated that the CRISPR-Cas system partially targets the *hutT* gene. Previous studies have shown that *hutT* activity is generally co-regulated with *hutH* (34), suggesting that the expression of *hutT* may influence the expression of other genes within the hut cluster. Contrary to our findings, previous studies have demonstrated that the *Klebsiella pneumoniae* ATCC 43186 strain can utilize histidine as a sole carbon source (40), potentially due to the absence of CRISPR spacers targeting the *hutT* gene. In contrast, the CRISPR-Cas system in strain Kp674 suppresses *hutT* mRNA expression via a spacer specifically binding to *hutT* DNA, thereby impairing the functionality of the entire *hut* gene cluster and reducing the ability to metabolize histidine as a carbon source for growth. This inference suggests that our findings may be strain dependent. Future studies should prioritize validating this regulatory mechanism across a broader range of strains to assess its universality in *Klebsiella pneumoniae* populations.

Studies have shown that the Hut system is not only associated with energy metabolism, but the *hut* genes are closely linked to bacterial virulence in many gram-negative pathogens (41–43). Based on our findings, we speculate that the absence of the CRISPR-Cas system in ∆*casABECD* strains leads to the increased expression of *hut* cluster genes, which could contribute to the observed reduction in the virulence of the strain. This aligns with prior research demonstrating that the overexpression of histidine utilization genes in *Pseudomonas aeruginosa* mutants results in reduced cytotoxicity toward macrophages, impaired T3SS effector protein transcription, and decreased virulence (41). The Hut system of *Acinetobacter baumannii* is linked to host zinc homeostasis. During zinc starvation, histidine catabolism mediated by *hutH* may release zinc from histidine–zinc complexes, thereby aiding bacterial survival in the host’s low-zinc environment induced by calprotectin (44). In contrast, *K. pneumoniae* exhibits high sensitivity to zinc toxicity, where excessive zinc inhibits its growth and virulence, indicating distinct survival strategies compared to *A. baumannii* (45). Additionally, a prospective study suggests that urocanate, an intermediate in the histidine degradation pathway, may accumulate in host tissues such as the skin and liver, serving as a potential signaling molecule for bacterial recognition of eukaryotic hosts and influencing bacterial pathogenicity and tissue dissemination (46).

This study contributes to understanding the role of the CRISPR-Cas system in upregulating bacterial virulence by targeting endogenous histidine utilization systems in *K. pneumoniae*. However, there are several limitations that may affect our interpretation of the CRISPR-Cas system’s impact on bacterial virulence. First, the challenges in purifying the Cascade complex hindered *in vitro* molecular experiments, limiting our ability to precisely explore the function of the CRISPR-Cas system mechanism in virulence regulation. Second, although a global gene analysis was performed, only the most significant gene clusters were selected for further investigation, potentially overlooking other important genes or regulatory networks involved in virulence. Additionally, while the *G. mellonella* model is widely employed in bacterial pathogenesis research, its lack of mammalian-like adaptive immunity limits its ability to assess hypervirulence dependent on complex host–pathogen interactions. Furthermore, although the function of histidine utilization genes has been explored, their exact role in bacterial virulence remains insufficiently investigated in *K. pneumoniae*, and the relationship between them is still unclear.

### Conclusions

**Fig 7:**
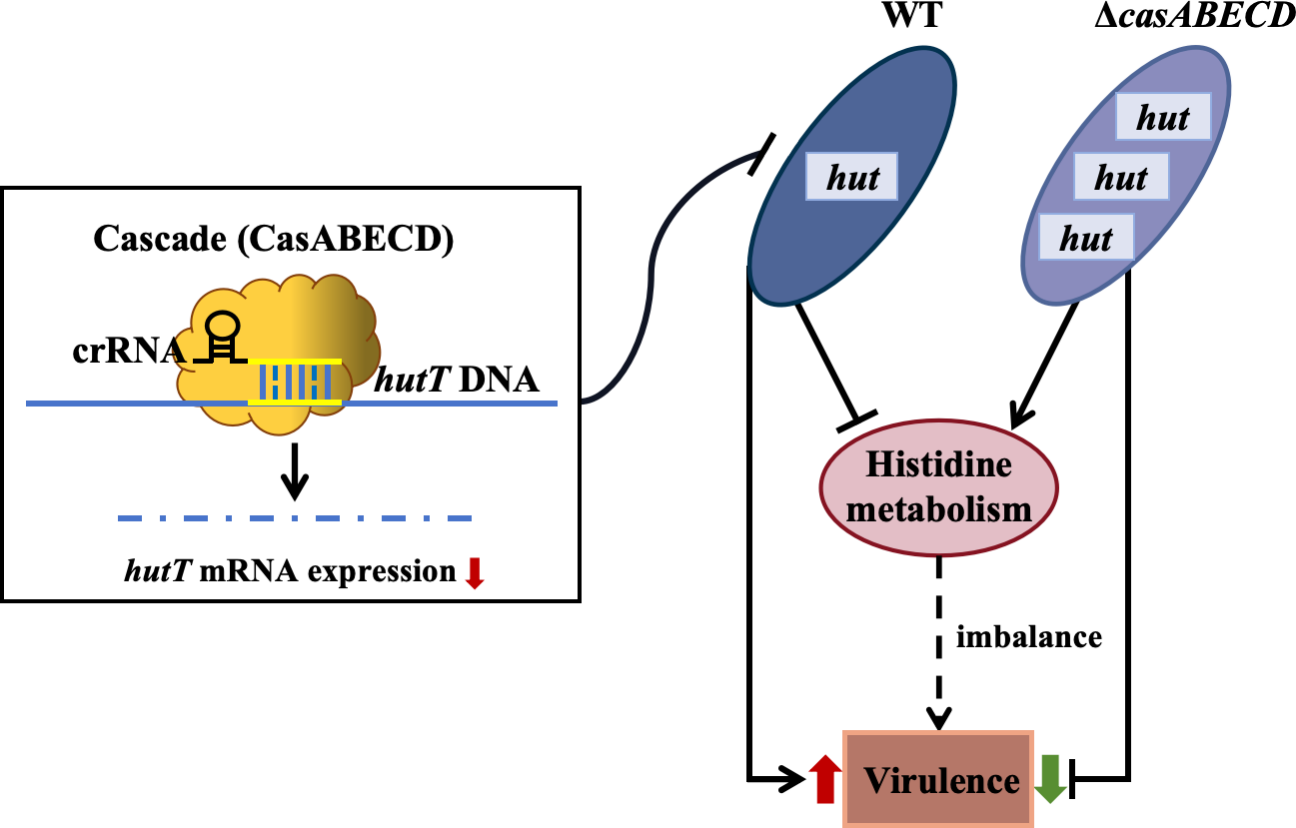
Proposed model of enhanced virulence in *K. pneumoniae* mediated by CRISPR-Cas targeting of *hutT*. The crRNA structure, associated with the Cascade (CasABECD complex), interacts with *hutT* through a sequence complementary to a part of the crRNA (highlighted in yellow). This interaction leads to the downregulation of *hutT* expression. Consequently, the reduced expression of *hutT* affects other *hut* genes within the same operon, thereby impacting histidine metabolism and enhancing bacterial virulence.

This study provides experimental evidence that the type I-E* CRISPR-Cas system in *Klebsiella pneumoniae* plays a crucial role in modulating bacterial virulence. The deficiency of the CRISPR-Cas system leads to significantly higher expression of the *hut* genes, which may cause an imbalance in histidine metabolism. This metabolic imbalance may impair bacterial fitness and contribute to the observed decrease in virulence of *K. pneumoniae* (Fig. 7). In summary, our findings indicate that the CRISPR-Cas system in *K. pneumoniae* regulates virulence through metabolic pathway modulation. Further exploration into the precise mechanisms by which the histidine utilization system influences virulence is needed to deepen our understanding of *K. pneumoniae* pathogenicity.

## MATERIALS AND METHODS

### Bacterial strains, plasmids, primers, and growth conditions

The bacterial strains and plasmids used in this study are summarized in Table S3, while all primers are detailed in Table S4. Polymerase chain reaction (PCR) was performed using forward and reverse primers designed to amplify the targeted deletion regions. Successful mutant construction was confirmed when the DNA product was amplified in the WT strain but not in the mutant strain. All bacterial strains were cultured at 37°C in LB broth or on LB agar supplemented with appropriate antibiotics. The wild-type *Klebsiella pneumoniae* Kp674 strain, a clinical isolate belonging to the ST23 and K1 capsular serotype, was maintained in LB broth.

### Construction of *cas* gene deletion strain ∆*cas* and *cas* gene complementary strain ∆*cas*/pBAD-*cas*

Deletion mutants were generated using lambda red recombination and allelic exchange with the suicide vector pKOBEG-Apra, as previously described (47). The ∆*casABECD* complemented mutant strain was constructed by amplifying the entire *casABECD* gene cluster (*casA*, *casB*, *casE*, *casC*, *casD*) from the genomic DNA of the WT strain. The amplified product was inserted into the pBAD33-Apra vector Seamless Cloning Kit (Vazyme Biotech, China). For complementation, competent cells of the isolate prepared according to standard methods and recombinant plasmids were introduced by electroporation (48). The resulting plasmids were introduced into the ∆*casABECD* deletion strain, and transformants were selected on LB agar supplemented with apramycin. Positive colonies were verified by PCR using primers targeting both the *casABECD* gene and the pBAD33-Apra plasmid (Table S4). Deletion or overexpression mutants for other genes in this study were also constructed using the methods described above.

### Determination of standard growth curve

The standard growth curve was established using a multifunctional enzyme marker (BioTek, Synergy H1, USA) as previously described (49). Each strain was cultured to an optical density at 600 nm (OD_600_) of 1.0 ± 0.02, and the bacterial suspension was subsequently diluted 100-fold in LB broth. Three independent biological replicates were conducted for each time point, and the data are presented as mean ± standard deviation (SD).

### Biofilm assays

Biofilm formation in LB broth was assessed and visualized following a previously described protocol with modifications (23, 35). The biofilm-forming abilities of the WT, Δ*casABECD*, and C-*casABECD* strains were assessed visually under static conditions after 72 h of incubation in LB broth at 37°C. Biofilms were stained with 1% crystal violet, washed with distilled water, and dissolved in 33% glacial acetic acid. The OD_590_ was measured to quantify the biofilm.

### Mucoviscosity assay

Bacterial cultures were grown to an OD_600_ of 1.0 ± 0.02, and 2 mL of each suspension was subjected to centrifugation at 2,500 × *g* for 5 min. Following centrifugation, 200 µL of the supernatant was carefully collected without disturbing the pellet, and the OD_600_ was measured to quantify the mucoviscosity (50).

### Iron acquisition testing

Overnight cultures of *K. pneumoniae* strains grown in LB broth were subcultured to an OD_600_ of 1.0 in LB broth supplemented with 200 µg/mL 2,2’-dipyridyl for iron starvation. Subsequently, 100 µL of the bacterial suspension was spotted onto agar plates containing Chrome azurol S (CAS)-iron(III)-hexadecyltrimethylammonium bromide (Qingdao Hope Biotech, China) and was incubated overnight at 37°C. The presence of an orange halo around the colony indicated siderophore production (51). Each strain was tested in triplicate.

For quantitative siderophore determination (52), a sterile culture supernatant was mixed 1:1 with CAS detection solution (Bioisco Biotech, China) and was incubated in the dark for 2 h before measuring the OD_630_, the absorbance at 630 nm (value A). A sterile culture medium mixed 1:1 with CAS solution was used as a control, incubated for the same duration, and its OD_630_ absorbance (value Ar) was recorded. The siderophore unit (SU) was calculated using the formula: SU = 1 A/Ar (52).

### Cell cytotoxicity assay

Cell cytotoxicity was evaluated by measuring the LDH release. A549 non-small lung carcinoma cells (ATCC) were seeded in 96-well plates at a density of 2 × 10^4^ cells per well and were cultured overnight. The following day, the cells were washed twice with PBS and then cultured in phenol red-free Dulbecco's modified Eagle's medium (DMEM). Bacteria were added at an MOI of 100:1 (bacteria-to-cell ratio) and were centrifuged at 300 × *g* for 5 min at room temperature to promote bacterial contact with the cells. After 4 h of incubation, the LDH levels in the cell culture supernatants were measured using the CytoTox 96 Non-Radioactive Cytotoxicity Assay kit (Promega Corporation, USA), following the manufacturer’s protocol. The degree of LDH release served as an indicator of the damage inflicted on A549 cells by the different bacterial strains.

### *Galleria mellonella* larvae infection

Virulence was assessed by infecting *Galleria mellonella* larvae as described previously, with slight modifications (7). Bacterial cells from the wild-type and mutant strains were collected by centrifugation at 3,000 × *g* for 5 min, washed twice with PBS, and resuspended to a final concentration of 5 × 10^7^ cells/mL. Each *G. mellonella* larva (*n* = 10 per group) was injected with 10 µL of the suspension, delivering an infection dose of 5 × 10^5^ cells. The survival of larvae was monitored every 24 h postinfection, and experiments were performed in triplicate. Survival curves were generated using GraphPad Prism software (San Diego, CA, USA), and statistical differences in survival rates between groups were evaluated using the log-rank (Mantel-Cox) test (53).

For the determination of the LD_50_, *G. mellonella* larvae (*n* = 10 per group) were injected with 10-fold serial dilutions of bacterial suspensions, containing 10^4^ to 10^6^ cells in PBS. Data from three independent experiments were pooled, and LD_50_ values were calculated using the Reed and Muench method (54).

### RNA isolation and qRT-PCR

RNA was extracted from bacterial cultures collected at the logarithmic growth phase. The total RNA of bacteria was extracted by using a Bacteria RNA Extraction Kit (Vazyme Biotech, China). A total of 50 ng of DNA-free RNA was used for first-strand cDNA synthesis with the HiScript III RT SuperMix for qPCR (+gDNA wiper) (Vazyme Biotech, China). qRT-PCR was performed using ChamQ Universal SYBR qPCR Master Mix (Vazyme Biotech, China) and gene-specific primers (Sangon Biotech, China) on a Real-Time PCR Detection System (Analytik Jena, qTOWER 3G touch, Germany). Relative transcript levels of individual genes were calculated using the 2^−∆∆CT^ method, with normalization to 16S rRNA levels. Primers used in qRT-PCR are listed in Table S4.

### Transcriptome analysis by RNA seq

Transcriptome analysis was conducted to compare gene expression profiles between the WT and Δ*casABECD* strains. Cultures of both strains were harvested at the logarithmic growth phase, with three biological replicates for each sample. Total RNA was extracted, and RNA sequencing (RNA seq) was performed using the Illumina platform at Shanghai Majorbio Bio-pharm Technology Biotech. Genes with adjusted *P* < 0.05, identified by DESeq, were considered differentially expressed (55). According to the KEGG and GO analysis, differentially significant genes were assigned to different functional groups. The data were analyzed on the online tool of Majorbio Cloud Platform (56). The heatmap was plotted by SRplot platform (57).

### Bioinformatic prediction of CRISPR-Cas system and analysis of crRNA targets

The CRISPR array and *cas* genes in the CRISPR-Cas system and intragenomic self-targeting sequences in Kp674 were analyzed using the online tools CRISPRCasFinder (https://crisprcas.i2bc.paris-saclay.fr/CrisprCasFinder/Index) (58) and CRISPRTarget (http://crispr.otago.ac.nz/CRISPRTarget/crispr_analysis.html) (59).

### Plasmid invasion assay and transcriptional validation

To evaluate CRISPR-Cas interference against spacer-targeted sequences, a plasmid-borne invader assay was conducted by comparing the transformation efficiency of targeted genes between the WT and ∆*casABECD* strains, as previously described (13, 60). The WT strain and the ∆*casABECD* mutant strain were transformed with either the *hutT*-harboring plasmid (pBAD33-Apra-*hutT*, p-*hutT*) or an empty control plasmid (empty vector, EV), using electroporation at a plasmid concentration of 100 ng under consistent electroporation conditions (Ec2, 2.4 kV, 4 ms) (Bio-Rad, MicroPulser, USA). Transformants were cultured in LB medium supplemented with 60 µg/mL apramycin. The relative plasmid transformation efficiency was calculated by comparing the number of transformants in the p-*hutT* group to the EV group, with the EV group serving as the baseline. Non-targeting CRISPR controls utilized p-*hutH* (pBAD33-Apra-*hutH*) harboring *hutH* unrelated to the CRISPR spacer. To further validate whether the Kp674 CRISPR-Cas system represses exogenous *hutT* gene expression, transcript levels of *hutT* (spacer targeted) and *hutH* (non-targeted) in p-*hutT*/p-*hutH*-transformed WT and Δ*casABECD* were quantified by qRT-PCR with 16S rRNA normalization and WT-EV as the baseline.

### Bacterial growth response to varying *L*-histidine levels in minimal medium

Bacterial cultures were grown in LB broth to an OD_600_ of approximately 1.0, then diluted 1:100 in M9 minimal medium containing *L*-histidine (*L*-His) at concentrations of 0, 2, 4, 8, 16, 32, 64, 128, and 256 mM (Aladdin Biotech, China). For controls, M9 medium was supplemented with either 0.4% glucose (Aladdin Biotech, China) or 10 mM *L*-glutamate (*L*-Glu) (Aladdin Biotech, China)*,* with growth monitored under identical conditions. The cultures were incubated at 37°C with shaking, and the growth of the WT and different mutant strains was monitored over time by measuring the OD_600_ at regular intervals to generate growth curves using a multifunctional enzyme marker (BioTek, Synergy H1, USA).

### Plasmid stability assay

To assess whether the pBAD33-Apra-*casABECD* plasmid in the C-*casABECD* strain is prone to loss in the medium when histidine is used as the sole carbon source compared to the standard LB medium, plasmid stability assays were performed with minor modifications following a previously described method (61). The C-*casABECD* strain was incubated on the LB agar plate containing 60 µg/mL apramycin at 37°C overnight. Thereafter, a single colony from the plate was picked and cultivated overnight at 37°C in 5 mL of LB broth. The overnight culture was then diluted 1:100 in 1 mL of fresh LB broth (without apramycin) or M9 minimal broth containing 256 mM *L*-His for titration. The cultures were subsequently re-grown at 37°C. To assess plasmid loss, 100 µL of the cultures collected from the tube after 24 h was plated on fresh LB agar with or without apramycin after serial dilution. Finally, the colonies on both plates were counted. The plasmid-containing fraction was calculated as follows: plasmid-containing fraction (%) = (number of colonies on antibiotic LB agar / number of colonies on non-antibiotic LB agar) × 100% (61).

### Statistical analysis

Statistical analyses were conducted using GraphPad Prism 7 software (San Diego, CA, USA) to calculate the mean ± SD and to determine the significance using one-way analysis of variance (ANOVA) with Dunnett’s multiple comparison tests comparing the WT and mutant strains. LD_50_ values were calculated using the Reed and Muench method (54). Survival curves were plotted using Kaplan-Meier, and group differences were analyzed by the log-rank (Mantel-Cox) test (53).

## Data Availability

RNA-seq data were deposited in the NCBI Sequence Read Archive (SRA) database under the BioProject accession number PRJNA1201895.

## SUPPLEMENTAL MATERIAL

Supplemental Tables msphere.00215-25-s0001.docx Tables S1 to S4.

ASM does not own the copyrights to Supplemental Material that may be linked to, or accessed through, an article. The authors have granted ASM a non-exclusive, world-wide license to publish the Supplemental Material files. Please contact the corresponding author directly for reuse.

## References

[1] Fu Y, Chen Y, Wang Y, Yao B, Li P, Yu Y. 2024. Susceptibility of various Gram-negative bacteria to antibacterial agents: SMART in China 2019-2020. BMC Microbiol 24:524. doi:10.1186/s12866-024-03526-8 39695970 PMC11656906

[2] Thomas AR, Candace MM. 2019. Hypervirulent Klebsiella pneumoniae. Clin Microbiol Rev 32:e00001-19. doi:10.1128/CMR.00001-19 31092506 PMC6589860

[3] Wyres KL, Lam MMC, Holt KE. 2020. Population genomics of Klebsiella pneumoniae. Nat Rev Microbiol 18:344–359. doi:10.1038/s41579-019-0315-1 32055025

[4] Podschun R, Ullmann U. 1998. Klebsiella spp. as nosocomial pathogens: epidemiology, taxonomy, typing methods, and pathogenicity factors. Clin Microbiol Rev 11:589–603. doi:10.1128/CMR.11.4.589 9767057 PMC88898

[5] Wang Q, Wang X, Wang J, Ouyang P, Jin C, Wang R, Zhang Y, Jin L, Chen H, Wang Z, et al.. 2018. Phenotypic and genotypic characterization of carbapenem-resistant Enterobacteriaceae: data from a longitudinal large-scale cre study in China (2012-2016). Clin Infect Dis 67:S196–S205. doi:10.1093/cid/ciy660 30423057

[6] Wyres KL, Wick RR, Judd LM, Froumine R, Tokolyi A, Gorrie CL, Lam MMC, Duchêne S, Jenney A, Holt KE. 2019. Distinct evolutionary dynamics of horizontal gene transfer in drug resistant and virulent clones of Klebsiella pneumoniae. PLoS Genet 15:e1008114. doi:10.1371/journal.pgen.1008114 30986243 PMC6483277

[7] Gu D, Dong N, Zheng Z, Lin D, Huang M, Wang L, Chan EW-C, Shu L, Yu J, Zhang R, Chen S. 2018. A fatal outbreak of ST11 carbapenem-resistant hypervirulent Klebsiella pneumoniae in a Chinese hospital: a molecular epidemiological study. Lancet Infect Dis 18:37–46. doi:10.1016/S1473-3099(17)30489-9 28864030

[8] Pu D, Zhao J, Chang K, Zhuo X, Cao B. 2023. “Superbugs” with hypervirulence and carbapenem resistance in Klebsiella pneumoniae: the rise of such emerging nosocomial pathogens in China. Sci Bull (Beijing) 68:2658–2670. doi:10.1016/j.scib.2023.09.040 37821268

[9] Horvath P, Barrangou R. 2010. CRISPR/Cas, the immune system of bacteria and archaea. Science 327:167–170. doi:10.1126/science.1179555 20056882

[10] Le C, Ann F, David R, Shuailiang C, Robert L, Naomi B, Patrick D H, H, Xuebing W, Wenyan J, Feng Z. 2013. Multiplex genome engineering using CRISPR/Cas systems. Science 339 10.1126/science.1231143 PMC3795411 23287718

[11] Mohanraju P, Saha C, van Baarlen P, Louwen R, Staals RHJ, van der Oost J. 2022. Alternative functions of CRISPR-Cas systems in the evolutionary arms race. Nat Rev Microbiol 20:351–364. doi:10.1038/s41579-021-00663-z 34992260

[12] Sampson TR, Saroj SD, Llewellyn AC, Tzeng YL, Weiss DS. 2013. A CRISPR/Cas system mediates bacterial innate immune evasion and virulence. Nature 497:254–257. doi:10.1038/nature12048 23584588 PMC3651764

[13] Li R, Fang L, Tan S, Yu M, Li X, He S, Wei Y, Li G, Jiang J, Wu M. 2016. Type I CRISPR-Cas targets endogenous genes and regulates virulence to evade mammalian host immunity. Cell Res 26:1273–1287. doi:10.1038/cr.2016.135 27857054 PMC5143421

[14] Shabbir MAB, Tang Y, Xu Z, Lin M, Cheng G, Dai M, Wang X, Liu Z, Yuan Z, Hao H. 2018. The involvement of the Cas9 gene in virulence of Campylobacter jejuni. Front Cell Infect Microbiol 8:285. doi:10.3389/fcimb.2018.00285 30177957 PMC6109747

[15] McGinn J, Marraffini LA. 2019. Molecular mechanisms of CRISPR-Cas spacer acquisition. Nat Rev Microbiol 17:7–12. doi:10.1038/s41579-018-0071-7 30171202

[16] Stern A, Keren L, Wurtzel O, Amitai G, Sorek R. 2010. Self-targeting by CRISPR: gene regulation or autoimmunity? Trends Genet 26:335–340. doi:10.1016/j.tig.2010.05.008 20598393 PMC2910793

[17] Sampson TR, Napier BA, Schroeder MR, Louwen R, Zhao J, Chin CY, Ratner HK, Llewellyn AC, Jones CL, Laroui H, Merlin D, Zhou P, Endtz HP, Weiss DS. 2014. A CRISPR-Cas system enhances envelope integrity mediating antibiotic resistance and inflammasome evasion. Proc Natl Acad Sci USA 111:11163–11168. doi:10.1073/pnas.1323025111 25024199 PMC4121812

[18] Aklujkar M, Lovley DR. 2010. Interference with histidyl-tRNA synthetase by a CRISPR spacer sequence as a factor in the evolution of Pelobacter carbinolicus. BMC Evol Biol 10:230. doi:10.1186/1471-2148-10-230 20667132 PMC2923632

[19] Shen J, Lv L, Wang X, Xiu Z, Chen G. 2017. Comparative analysis of CRISPR-Cas systems in Klebsiella genomes. J Basic Microbiol 57:325–336. doi:10.1002/jobm.201600589 28156004

[20] Kadkhoda H, Gholizadeh P, Ghotaslou R, Pirzadeh T, Ahangarzadeh Rezaee M, Nabizadeh E, Feizi H, Samadi Kafil H, Aghazadeh M. 2024. Prevalence of the CRISPR-cas system and its association with antibiotic resistance in clinical Klebsiella pneumoniae isolates. BMC Infect Dis 24:554. doi:10.1186/s12879-024-09451-5 38831286 PMC11149351

[21] Owaid HA, Al-Ouqaili MTS. 2024. Molecular and bacteriological investigations for the co-existence CRISPR/Cas system and β-lactamases of types extended-spectrum and carbapenemases in Multidrug, extensive drug and Pandrug-Resistant Klebsiella pneumoniae. Saudi J Biol Sci 31:104022. doi:10.1016/j.sjbs.2024.104022 38817398 PMC11137337

[22] Paczosa MK, Mecsas J. 2016. Klebsiella pneumoniae: going on the offense with a strong defense. Microbiol Mol Biol Rev 80:629–661. doi:10.1128/MMBR.00078-15 27307579 PMC4981674

[23] Cubero M, Marti S, Domínguez MÁ, González-Díaz A, Berbel D, Ardanuy C. 2019. Hypervirulent Klebsiella pneumoniae serotype K1 clinical isolates form robust biofilms at the air-liquid interface. PLoS One 14:e0222628. doi:10.1371/journal.pone.0222628 31532800 PMC6750583

[24] Walker KA, Miller VL. 2020. The intersection of capsule gene expression, hypermucoviscosity and hypervirulence in Klebsiella pneumoniae. Curr Opin Microbiol 54:95–102. doi:10.1016/j.mib.2020.01.006 32062153 PMC8121214

[25] Mendes G, Santos ML, Ramalho JF, Duarte A, Caneiras C. 2023. Virulence factors in carbapenem-resistant hypervirulent Klebsiella pneumoniae. Front Microbiol 14:1325077. doi:10.3389/fmicb.2023.1325077 38098668 PMC10720631

[26] Zhou Y, Wu X, Wu C, Zhou P, Yang Y, Wang B, Xu Y, Zhao H, Guo Y, Yu J, Yu F. 2024. Emergence of KPC-2 and NDM-5-coproducing hypervirulent carbapenem-resistant Klebsiella pneumoniae with high-risk sequence types ST11 and ST15. mSphere 9:e00612-23. doi:10.1128/msphere.00612-23 38193656 PMC10826354

[27] Zhou Y, Wu C, Wang B, Xu Y, Zhao H, Guo Y, Wu X, Yu J, Rao L, Wang X, Yu F. 2023. Characterization difference of typical KL1, KL2 and ST11-KL64 hypervirulent and carbapenem-resistant Klebsiella pneumoniae. Drug Resist Updat 67:100918. doi:10.1016/j.drup.2023.100918 36610180

[28] Bender RA. 2012. Regulation of the histidine utilization (hut) system in bacteria. Microbiol Mol Biol Rev 76:565–584. doi:10.1128/MMBR.00014-12 22933560 PMC3429618

[29] Schroll C, Barken KB, Krogfelt KA, Struve C. 2010. Role of type 1 and type 3 fimbriae in Klebsiella pneumoniae biofilm formation. BMC Microbiol 10:179. doi:10.1186/1471-2180-10-179 20573190 PMC2911432

[30] Mächtel R, Narducci A, Griffith DA, Cordes T, Orelle C. 2019. An integrated transport mechanism of the maltose ABC importer. Res Microbiol 170:321–337. doi:10.1016/j.resmic.2019.09.004 31560984 PMC6906923

[31] Sinha R, LeVeque RM, Callahan SM, Chatterjee S, Stopnisek N, Kuipel M, Johnson JG, DiRita VJ. 2023. Gut metabolite L-lactate supports Campylobacter jejuni population expansion during acute infection. bioRxiv:2023.10.02.560557. doi:10.1101/2023.10.02.560557 PMC10786315 38170751

[32] Zhang J, Xu Y, Wang M, Li X, Liu Z, Kuang D, Deng Z, Ou HY, Qu J. 2023. Mobilizable plasmids drive the spread of antimicrobial resistance genes and virulence genes in Klebsiella pneumoniae. Genome Med 15:106. doi:10.1186/s13073-023-01260-w 38041146 PMC10691111

[33] Schwacha A, Cohen JA, Gehring KB, Bender RA. 1990. Tn1000-mediated insertion mutagenesis of the histidine utilization (hut) gene cluster from Klebsiella aerogenes: genetic analysis of hut and unusual target specificity of Tn1000. J Bacteriol 172:5991–5998. doi:10.1128/jb.172.10.5991-5998.1990 2170334 PMC526921

[34] Schlesinger S, Magasanik B. 1965. Imidazolepropionate, a nonmetabolizable inducer for the histidine-degrading enzymes in Aerobacter aerogenes. J Biol Chem 240:4325–4330. 5845835

[35] Cui L, Wang X, Huang D, Zhao Y, Feng J, Lu Q, Pu Q, Wang Y, Cheng G, Wu M, Dai M. 2020. CRISPR-cas3 of Salmonella upregulates bacterial biofilm formation and virulence to host cells by targeting quorum-sensing systems. Pathogens 9:53. doi:10.3390/pathogens9010053 31936769 PMC7168661

[36] Solbiati J, Duran-Pinedo A, Godoy Rocha F, Gibson FC 3rd, Frias-Lopez J. 2020. Virulence of the pathogen Porphyromonas gingivalis is controlled by the CRISPR-Cas protein Cas3. mSystems 5:e00852-20. doi:10.1128/mSystems.00852-20 32994292 PMC7527141

[37] Liao W, Liu Y, Chen C, Li J, Du F, Long D, Zhang W. 2020. Distribution of CRISPR-Cas systems in clinical carbapenem-resistant Klebsiella pneumoniae strains in a Chinese tertiary hospital and its potential relationship with virulence. Microb Drug Resist 26:630–636. doi:10.1089/mdr.2019.0276 31834846

[38] Akashi H, Gojobori T. 2002. Metabolic efficiency and amino acid composition in the proteomes of Escherichia coli and Bacillus subtilis. Proc Natl Acad Sci USA 99:3695–3700. doi:10.1073/pnas.062526999 11904428 PMC122586

[39] Ingle RA. 2011. Histidine biosynthesis. Arabidopsis Book 9:e0141. doi:10.1199/tab.0141 22303266 PMC3266711

[40] Vornhagen J, Sun Y, Breen P, Forsyth V, Zhao L, Mobley HLT, Bachman MA. 2019. The Klebsiella pneumoniae citrate synthase gene, gltA, influences site specific fitness during infection. PLoS Pathog 15:e1008010. doi:10.1371/journal.ppat.1008010 31449551 PMC6730947

[41] Rietsch A, Wolfgang MC, Mekalanos JJ. 2004. Effect of metabolic imbalance on expression of type III secretion genes in Pseudomonas aeruginosa. Infect Immun 72:1383–1390. doi:10.1128/IAI.72.3.1383-1390.2004 14977942 PMC356022

[42] Lonergan ZR, Palmer LD, Skaar EP. 2020. Histidine utilization is a critical determinant of Acinetobacter pathogenesis. Infect Immun 88:e00118-20. doi:10.1128/IAI.00118-20 32341119 PMC7309604

[43] Sieira R, Arocena GM, Bukata L, Comerci DJ, Ugalde RA. 2010. Metabolic control of virulence genes in Brucella abortus: HutC coordinates virB expression and the histidine utilization pathway by direct binding to both promoters. J Bacteriol 192:217–224. doi:10.1128/JB.01124-09 19854911 PMC2798258

[44] Nairn BL, Lonergan ZR, Wang J, Braymer JJ, Zhang Y, Calcutt MW, Lisher JP, Gilston BA, Chazin WJ, de Crécy-Lagard V, Giedroc DP, Skaar EP. 2016. The response of Acinetobacter baumannii to zinc starvation. Cell Host Microbe 19:826–836. doi:10.1016/j.chom.2016.05.007 27281572 PMC4901392

[45] Maunders EA, Ganio K, Hayes AJ, Neville SL, Davies MR, Strugnell RA, McDevitt CA, Tan A. 2022. The role of ZntA in Klebsiella pneumoniae zinc homeostasis. Microbiol Spectr 10:e01773-21. doi:10.1128/spectrum.01773-21 35019689 PMC8754117

[46] Zhang X-X, Ritchie SR, Rainey PB. 2014. Urocanate as a potential signaling molecule for bacterial recognition of eukaryotic hosts. Cell Mol Life Sci 71:541–547. doi:10.1007/s00018-013-1527-6 24305948 PMC11113655

[47] Bi D, Jiang X, Sheng ZK, Ngmenterebo D, Tai C, Wang M, Deng Z, Rajakumar K, Ou HY. 2015. Mapping the resistance-associated mobilome of a carbapenem-resistant Klebsiella pneumoniae strain reveals insights into factors shaping these regions and facilitates generation of a “resistance-disarmed” model organism. J Antimicrob Chemother 70:2770–2774. doi:10.1093/jac/dkv204 26169555

[48] Dower WJ, Miller JF, Ragsdale CW. 1988. High efficiency transformation of E. coli by high voltage electroporation. Nucleic Acids Res 16:6127–6145. doi:10.1093/nar/16.13.6127 3041370 PMC336852

[49] Krishnamurthi VR, Niyonshuti II, Chen J, Wang Y. 2021. A new analysis method for evaluating bacterial growth with microplate readers. PLoS One 16:e0245205. doi:10.1371/journal.pone.0245205 33434196 PMC7802944

[50] Xu Y, Zhang J, Wang M, Liu M, Liu G, Qu H, Liu J, Deng Z, Sun J, Ou HY, Qu J. 2021. Mobilization of the nonconjugative virulence plasmid from hypervirulent Klebsiella pneumoniae. Genome Med 13:119. doi:10.1186/s13073-021-00936-5 34294113 PMC8299605

[51] Schwyn B, Neilands JB. 1987. Universal chemical assay for the detection and determination of siderophores. Anal Biochem 160:47–56. doi:10.1016/0003-2697(87)90612-9 2952030

[52] Gu S, Wan W, Shao Z, Zhong W. 2021. High-throughput method for detecting siderophore production by rhizosphere bacteria. Bio Protoc 11:e4001. doi:10.21769/BioProtoc.4001 PMC8161126 34124302

[53] Stempinski PR, Smith DFQ, Casadevall A. 2022. Cryptococcus neoformans virulence assay using a Galleria mellonella larvae model system. Bio Protoc 12. doi:10.21769/BioProtoc.4480 PMC9411016 36082366

[54] Insua JL, Llobet E, Moranta D, Pérez-Gutiérrez C, Tomás A, Garmendia J, Bengoechea JA. 2013. Modeling Klebsiella pneumoniae pathogenesis by infection of the wax moth Galleria mellonella. Infect Immun 81:3552–3565. doi:10.1128/IAI.00391-13 23836821 PMC3811777

[55] Wang L, Feng Z, Wang X, Wang X, Zhang X. 2010. DEGseq: an R package for identifying differentially expressed genes from RNA-seq data. Bioinformatics 26:136–138. doi:10.1093/bioinformatics/btp612 19855105

[56] Han C, Shi C, Liu L, Han J, Yang Q, Wang Y, Li X, Fu W, Gao H, Huang H, Zhang X, Yu K. 2024. Majorbio Cloud 2024: update single-cell and multiomics workflows. Imeta 3:e217. doi:10.1002/imt2.217 39135689 PMC11316920

[57] Tang D, Chen M, Huang X, Zhang G, Zeng L, Zhang G, Wu S, Wang Y. 2023. SRplot: a free online platform for data visualization and graphing. PLoS One 18:e0294236. doi:10.1371/journal.pone.0294236 37943830 PMC10635526

[58] Couvin D, Bernheim A, Toffano-Nioche C, Touchon M, Michalik J, Néron B, Rocha EPC, Vergnaud G, Gautheret D, Pourcel C. 2018. CRISPRCasFinder, an update of CRISRFinder, includes a portable version, enhanced performance and integrates search for Cas proteins. Nucleic Acids Res 46:W246–W251. doi:10.1093/nar/gky425 29790974 PMC6030898

[59] Biswas A, Gagnon JN, Brouns SJJ, Fineran PC, Brown CM. 2013. CRISPRTarget: bioinformatic prediction and analysis of crRNA targets. RNA Biol 10:817–827. doi:10.4161/rna.24046 23492433 PMC3737339

[60] Cass SDB, Haas KA, Stoll B, Alkhnbashi OS, Sharma K, Urlaub H, Backofen R, Marchfelder A, Bolt EL. 2015. The role of Cas8 in type I CRISPR interference. Biosci Rep 35:e00197. doi:10.1042/BSR20150043 26182359 PMC4613674

[61] Chen Y, Goh YX, Li P, Guan J, Chao Y, Qu H, Ou HY, Wang X. 2024. RES-Xre toxin-antitoxin locus knaAT maintains the stability of the virulence plasmid in Klebsiella pneumoniae. Emerg Microbes Infect 13:2316814. doi:10.1080/22221751.2024.2316814 38323903 PMC10896132

