# Supplementary material for: Type I-E* CRISPR-Cas of Klebsiella pneumoniae upregulates bacterial virulence by targeting endogenous histidine utilization system
Source: mSphere. 2025 May 19;10(6):e00215-25. doi: 10.1128/msphere.00215-25 (PMC12188710; doi:10.1128/msphere.00215-25)
Supplement: Supplemental Tables — Tables S1 to S4. [file msphere.00215-25-s0001.docx]

**Type I-E* CRISPR-Cas of *Klebsiella pneumoniae* Upregulates Bacterial Virulence by Targeting Endogenous Histidine Utilization Systems**

Supplementary data

Table S1. The information of spacers in Klebsiella pneumoniae Kp674 strain.

Table S2. Distribution of CRISPR-Cas spacers targeting the *hutT* gene across 1,194 *Klebsiella pneumoniae* strains.

Table S3. Strains and plasmids used in this study.

**Table S4. Oligonucleotides used in this study.**

Table S1. The information of spacers in Klebsiella pneumoniae Kp674 strain.

| **Location** | **Direct Repeat sequence** | **Spacer sequence** |
| --- | --- | --- |
| CRISPR array1 | GTCTTCCCCACACGCGTGGGGGTGTTTC | TATGGGTTGCAGTGTAAGAGCGGCAAACGAAGC |
|  |  | CGGCTCTTTTTTATCTCCTTCATCCTTCGCTAT |
|  |  | TGATCGGCGTGCCGTTTGTTGGACCCGAAATAG |
|  |  | CGTCATCAGCGCCTTGTTCCAGCGGCGACCACC |
|  |  | TATCGTGCAGAGTCACAACCTGACGGGATTATC |
|  |  | TCGTGCATGGTGAGGATTCTACAGTCGCACCAT |
|  |  | TACCTCCCGGCGTCCGCGCCAGGGCGATCACGTG |
|  |  | CCTGCAGCTGGCCGTCGAGCTGACGGATGCCGG |
|  |  | TTCATCACGTGTGAGCGGATTTGGCTCTATCCT |
| CRISPR array2 | GTCTTCCCCACGCACGTGGGGGTGTTTC | TGATCCCCCATTCGATTACCGAGTGGGCAGCGG |
|  |  | TGATAGCGGCAACAACTTTCCATTTAGCCTTGA |
|  |  | TAGCAGAAGCCTCAATGTCGATAGGTGAGTGTC |
|  |  | CAATTTGACCGCTTGCGAAGAGTATTTTGCATG |
|  |  | TAAATGCAACGAATGGTTTACAATGGATGTTGA |
|  |  | CGATGTATTCTATGATGACATCATGGCTTCGCA |
|  |  | CCACATGCCATCATGAGTGCCTGACATGGCATA |
|  |  | TAAGCCTTACACTTCCGTACTGAAAAGCGGATT |
|  |  | TCAGCGCATCATTGGCTACACCGGTAAGGGTGT |
|  |  | CCCTTTGAGGCGCACATAAGCCTTTGCACGCTC |
|  |  | TCAGATGAAGCTACTCGATACGGCAGACTGCAC |
|  |  | TATTTCAATTTCGGCTCAAAAGGATGCTGTGAG |
|  |  | TAGAACAAAGCGAGGGGTGCCTGGGGATGAAGT |
|  |  | CCACCCGAACACCGTGCAGCTCAACCTGAGCAC |

Table S1 (Continued). The information of spacers in *Klebsiella pneumoniae* Kp674 strain.

| **Location** | **Direct Repeat sequence** | **Spacer sequence** |
| --- | --- | --- |
| CRISPR array2 | GTCTTCCCCACGCACGTGGGGGTGTTTC | TTTGCAACAATATACCACTTCCCGACCTTTGAT |
|  |  | TAGGTTTTTGTTGTCTGATTGAATGGGTTGTCA |
|  |  | TGCTTCTATTAACCTGTTTCTGTAGCTTAAACC |
|  |  | TGATGTGGGAGTGTGGTGTAAAAAACTCTCTTT |
|  |  | TCTTATGCACGGTGTTGGATACGAACCACTCCA |
|  |  | TACCCCATTTCTGTCTTTTGTAAGCTCCATCAC |
|  |  | TATTAATGCTTACGTTTCGTGGTTTGAGGATGA |
|  |  | CGTCGTGAATTACGGCAATACGCTGGACCGCAA |
|  |  | CAGTACCGCCAGCAGCGCCTTGATGCCATGTGG |
|  |  | TTAACTATGCAGCGTAACCGTGCTTTCGTCTCA |
|  |  | CTGCAAAAGCAAAATGTTGCTCTGTCAGGTGCA |
|  |  | TATCAATCTCTGAAATCATTTTGTCTATTTTGT |
|  |  | CGCGACGACCTCGTCATCCTCCCCGCTGCGGCT |
|  |  | TACACCCAGCTCTTTGACGCAAAGGCTCAGGAG |
|  |  | TCCTTGGTTCGTTTCTTCTGATGTTTGCAAGGC |
|  |  | CTGGTTGACGTATGCCGTGATGCTGCTGGTAGG |
|  |  | CATTCGAGTCAGTAAAAGCGTCTAGTGCTGACG |
|  |  | CGCTGTATGCCCCCCATCCTTCGCAAGCACTAC |
|  |  | CGATGACAACTTTGCCTACTTCGCTGCGCTGGC |
|  |  | TTCCTCTTCTTCCTGACAATCAGCGCAGCGCTG |
|  |  | ***TGCGGGTGGATGACAATAACGCCTGGCGCGCCG*** |
|  |  | CCTGTTAAGGATGACGCGGGGAAATGGGTTATC |
|  |  | TCGATGGCGAGCTGCTGGTGAAAAAGTCGATAC |
|  |  | TCCAGGCCAGATCTGTTTGGGTTGGTGTCGAAG |
|  |  | TGAAGGTGAGCGCCAAAAGGAGTGGAAGCATGC |
|  |  | CGGCTGCGTTATCCGGCAGCACGAACTGGACCT |
|  |  | TGACCTTAGAGGCTTCACTCAGTGCCACTTTTT |

Note: The spacer targeting *hutT* was bolded and italicized

Table S2. Distribution of CRISPR-Cas spacers targeting the *hutT* gene across 1,194 *Klebsiella pneumoniae* strains.

| **Spacer sequence** | **Accession no.** | **Location** | **GenBank assembly accession** | **MLST type** |
| --- | --- | --- | --- | --- |
| TGCGGGTGGATGACAATAACGCCTGGCGCGCCG | CP030269.1 | Chromosome | GCA_003286995.1 | ST1660 |
|  | CP025641.1 | Chromosome | GCA_002870945.1 | ST1941 |
|  | CP003785.1 | Chromosome | GCA_000294365.1 | ST23 |
|  | CP016813.1 | Chromosome | GCA_001708245.1 | ST23 |
|  | CP025631.1 | Chromosome | GCA_002870905.1 | ST23 |
|  | CP025633.1 | Chromosome | GCA_002870925.1 | ST23 |
|  | CP027189.1 | Chromosome | GCA_002970895.1 | ST23 |
|  | CP037742.1 | Chromosome | GCA_004924315.1 | ST23 |
|  | CP047595.1 | Chromosome | GCA_018279185.1 | ST23 |
|  | CP052562.1 | Chromosome | GCA_012970645.1 | ST23 |
|  | CP067060.1 | Chromosome | GCA_019454345.1 | ST23 |
|  | CP068015.1 | Chromosome | GCA_018604345.1 | ST23 |
|  | CP082805.1 | Chromosome | GCA_019915305.1 | ST23 |
|  | LR890464.1 | Chromosome | GCA_904864645.1 | ST23 |
| CGGCGCGCCAGGCGTTATTGTCATCCACCCGCA (reverse complement) | AP006725.1 | Chromosome | GCA_000009885.1 | ST23 |
|  | CP025088.1 | Chromosome | GCA_002845965.1 | ST1941 |
|  | CP014010.1 | Chromosome | GCA_001529935.1 | ST23 |
|  | CP025087.1 | Chromosome | GCA_002831525.1 | ST23 |
|  | CP047675.1 | Chromosome | GCA_009909325.1 | ST23 |
|  | CP063890.1 | Chromosome | GCA_015284665.1 | ST23 |
|  | CP063908.1 | Chromosome | GCA_015286445.1 | ST23 |

Source: Zhang, et al., Genome Med. 2023 (PMID: 38041146)

Table S3. Strains and plasmids used in this study.

| **Strain/plasmid** | **Description** | **Source or Reference** |
| --- | --- | --- |
| *K. pneumoniae* Kp674 (WT) | ST23 and K1 capsular serotype | This study |
| *K. pneumoniae* Δ*casABECD* | the *casABECD* genes of Kp674 were deleted | This study |
| *K. pneumoniae* C-*casABECD* | the *casABECD* genes of Δ*casABECD* were complemented;  full name: Δ*casABECD*/pBAD33-Apra-*casABECD* | This study |
| *K. pneumoniae* Δ*casABECD*/Δ*hutH* | the *casABECD* and *hutH* genes of Kp674 were deleted | This study |
| *K. pneumoniae* WT/p-*hutT* | the *hutT gene* of Kp674 was overexpressed | This study |
| pKOBEG-Apra | Thermo-sensitive replicon (growth at 30 °C); λ red genes *gam*, *bet* and *exo* under pBAD promoter (arabinose inducible); Apra^R^ | Chaveroche, *et al*., Nucleic Acids Res, 2000 (PMID: 11071951) |
| pJTAG-Hyg | pJTAG-derived; flippase recognition target (FRT)-flanked *hph*cassette; Hyg^R^ | Kochar, *et al*., Antimicrob Agents Chemother, 2012 (PMID: 22290963) |
| pFLP2-Apra | pFLP2-derived; *sacB flp cI*; Apra^R^ | Hoang, *et al*., Gene, 1998 (PMID: 9661666) |
| pBAD33-Apra | p15A ori; araC; P_ara_ promoter, Apra^R^ | Liu, *et al*., Front Cell Infect Microbiol, 2017 (PMID: 29085808) |

**Table S4. Oligonucleotides used in this study.**

| **Name** | **Sequence (5’-3’)** | **Description** |
| --- | --- | --- |
| **Homologous Recombination Primers** | | |
| EBGNHe-5 | CCCGCTAGCGAAAAGATGTTTCGTGAAGC | Validation of the pKOBEG plasmid; associated with λ red genes in pKOBEG-Apra |
| EBGh3-3 | GGGAAGCTTATTATCGTGAGGATGCGTCA |  |
| GmF | CGAATTAGCTTCAAAAGCGCTCTGA | Amplification of the *hph* cassette; derived from pJTAG-Hyg |
| GmR-2 | AATTGGGGATCTTGAAGTACCT |  |
| *hph*-F | CGCATAGACGTCGGTGAAGT | Validation of the hygromycin gene fragment |
| *hph*-R | GATGATTCCTACGCGAGCCT |  |
| PR1655 | TGCTCTAGAGCACGGCATTTTCTTTTGCGTTT | Validation of the pFLP2-Apra plasmid; associated with the *sacB* gene |
| PR1656 | CGCGGATCCGCGTCTTTAGGCCCGTAGTCTGC |  |
| *casABECD-*UF | GACACCCATGACCTGTTGCT | Amplification of upstream arm fragments of *casABECD* |
| *casABECD*-UR | tcagagcgcttttgaagctaattcgCGCAATCCAGGGTTCATCAAT |  |
| *casABECD*-DF | aggtacttcaagatccccaattGTGCTGGAGTGCGGCTAAT | Amplification of downstream arm fragments of *casABECD* |
| *casABECD*-DR | CGTTTAGCAAACATCGCCCG |  |
| *casABECD-F* | GGATTTCGCAGGGCTACTCA | Validation of the deletion of *casABECD* |
| *casABECD-R* | CAGCACTGCGATGACACTCT |  |
| *hutH-*UF | AAGGTATAGGTCCAGCCGGT | Amplification of upstream arm fragments of *hutH* |
| *hutH*-UR | tcagagcgcttttgaagctaattcgATAACGCCATTGCGCTTCTG |  |
| *hutH*-DF | aggtacttcaagatccccaattGCGACAGGCTGAGTTGAC | Amplification of downstream arm fragments of *hutH* |
| *hutH*-DR | TATCTGCTGGGTCGGTCTGG |  |
| **Complementation and Overexpression Primers** | | |
| pBAD-cF | GCATGCAAGCTTGGCTGTTT | Linear cloning of the pBAD33-Apra plasmid |
| pBAD-cR | GAGCTCGAATTCGCTAGCCC |  |
| pBAD-F | ATGCCATAGCATTTTTATCC | Validation of the pBAD33-Apra plasmid |
| pBAD-R | GATTTAATCTGTATCAGG |  |
| *casABECD*-Fc | gggctagcgaattcgagctcACCATGGAGAACCGCTTCAA | Cloning of *casABECD* |
| *casABECD*-Rc | aaacagccaagcttgcatgcCCATTAGCCGCACTCCAGC |  |

**Table S4** (Continued)**. Oligonucleotides used in this study.**

| **Name** | **Sequence (5’-3’)** | **Description** |
| --- | --- | --- |
| **Complementation and Overexpression Primers** | | |
| *hutT*-Fc | gggctagcgaattcgagctcATGCAACAACAACACAAGCCAC | Cloning of *hutT* |
| *hutT*-Rc | aaacagccaagcttgcatgcACCGGGGAAATTGATCTTACTG |  |
| *hutH*-Fc | gcgaattcgagctcAACGCAGGGGAACGCTAAA | Cloning of *hutH* |
| *hutH*-Rc | aaacagccaagcttgcatgcGACGCGGGTACAGAGAACAACG |  |
| **qRT-PCR Primers** | | |
| 16S rRNA-F | ACTCCTACGGGAGGCAGCAGT | Primers targeting the 16S rRNA gene, used as the reference gene for normalization |
| 16S rRNA-R | TATTACCGCGGCTGCTGGC |  |
| *hutT*-qF | CTGTTCGCCAAAGCTGTTCC | Primers for qRT-PCR amplification of the *hutT* gene |
| *hutT*-qR | GATCCCGCAGGCCATCAATA |  |
| *hutH*-qF | GACGTGGTCTTCCTGGTTGG | Validation of the deletion of *hutH* and primers for qRT-PCR amplification of the *hutH* gene |
| *hutH*-qR | GTAACTTCCATGCCGAACCG |  |
| *hutU*-qF | AGGTTGGAGTTGGCGATCAG | Primers for qRT-PCR amplification of the *hutU* gene |
| *hutU*-qR | GCGCAACTGGGAATGCTATG |  |
| *hutC*-qF | AGAAATTGTCGCCCGTCACCATC | Primers for qRT-PCR amplification of the *hutC* gene |
| *hutC*-qR | CGCCGTCGCCTGAATATGATCC |  |
| *hutG*-qF | CATGACCGCTGCGTGGATATGG | Primers for qRT-PCR amplification of the *hutG* gene |
| *hutG*-qR | TTACCCGCCCGCTGACAGTC |  |
| *hutI*-qF | GGCGATGCTGACGGCTATATTACG | Primers for qRT-PCR amplification of the *hutI* gene |
| *hutI*-qR | AACGCTTTCAAACAGTCCCTCCTG |  |
